# Nop17 is a key R2TP factor for the assembly and maturation of box C/D snoRNP complex

**DOI:** 10.1186/s12867-015-0037-5

**Published:** 2015-03-18

**Authors:** Marcela B Prieto, Raphaela C Georg, Fernando A Gonzales-Zubiate, Juliana S Luz, Carla C Oliveira

**Affiliations:** Department of Biochemistry, Institute of Chemistry, University of São Paulo, Av. Prof. Lineu Prestes 748, 05508-000 São Paulo, SP Brazil; Present address: Department of Biochemistry and Molecular Biology, Institute of Biological Sciences, Federal University of Goiás, Goiânia, Brazil; Present address: Department of Biological Sciences, School of Pharmacy, São Paulo State University, Araraquara, Brazil

**Keywords:** box C/D snoRNP assembly, R_2_TP complex, Nop17

## Abstract

**Background:**

Box C/D snoRNPs are responsible for rRNA methylation and processing, and are formed by snoRNAs and four conserved proteins, Nop1, Nop56, Nop58 and Snu13. The snoRNP assembly is a stepwise process, involving other protein complexes, among which the R_2_TP and Hsp90 chaperone. Nop17, also known as Pih1, has been shown to be a constituent of the R_2_TP (Rvb1, Rvb2, Tah1, Pih1) and to participate in box C/D snoRNP assembly by its interaction with Nop58. The molecular function of Nop17, however, has not yet been described.

**Results:**

To shed light on the role played by Nop17 in the maturation of snoRNP, here we analyzed the interactions domains of Nop58 – Nop17 – Tah1 and the importance of ATP to the interaction between Nop17 and the ATPase Rvb1/2.

**Conclusions:**

Based on the results shown here, we propose a model for the assembly of box C/D snoRNP, according to which R_2_TP complex is important for reducing the affinity of Nop58 for snoRNA, and for the binding of the other snoRNP subunits.

**Electronic supplementary material:**

The online version of this article (doi:10.1186/s12867-015-0037-5) contains supplementary material, which is available to authorized users.

## Background

Box C/D snoRNP complexes are involved in pre-rRNA cleavage and in 2′-O-methylation of nucleotides at specific positions in rRNAs, snRNAs, and other RNAs during maturation [1]. In yeast, these complexes are formed by snoRNAs that contain conserved sequences (boxes C, D, C’ and D’), and four core proteins, Nop1, Nop56, Nop58 and Snu13. In addition to these proteins, some other factors may associate with specific snoRNP, such as the U3 snoRNP [2]. snoRNP complexes are conserved from archaea to eukaryotes, although in the latter they are more complex [3].

The assembly of snoRNP is initiated in the nucleoplasm and completed in the cajal bodies in mammalian cells, whereas in yeast, the final steps of assembly and maturation of snoRNPs are considered to occur in the nucleolus, a compartment where the snoRNPs also catalyze the rRNA modifications [4,5].

During snoRNP assembly, Snu13 binds RNA by recognizing a conserved RNA secondary structure that is present in box C/D snoRNAs, as well as in the U4 snRNA [6]. Due to its affinity to RNA, the human orthologue of Snu13, 15.5kD, has been shown to be the first core snoRNP subunit to bind box C/D snoRNAs [7]. Although the later steps of assembly are less well defined, it has been shown that Nop1 and Nop58 bind the snoRNAs independently, whereas Nop56 depends on Nop1 for binding the complexes [3]. Due to the many structural rearrangements that occur during assembly, chaperones may be important for the maturation of snoRNPs.

Nop17, also known as Pih1, has been shown to strongly interact with Nop58 [8] and with the chaperone Hsp90 [9]. Through its interaction with Hsp90, Nop17 was identified as part of a complex named R_2_TP (Rvb1, Rvb2, Tah1, Pih1) [9,10]. Rvb1 and Rvb2 are ATP dependent helicases, belonging to the class of AAA^+^ ATPases [11], that form heterohexamers *in vitro* and participate in processes ranging from DNA repair, transcription, chromatin remodeling, ribosomal RNA processing, to small nucleolar RNP formation [12]. The human orthologues of Rvb1/Rvb2, TIP48/TIP49, have also been shown to be involved in box C/D snoRNP assembly [7].

Tah1, a TPR (tetratricopeptide repeat)-containing protein associated with heat-shock protein Hsp90 has been shown to bind directly to Hsp90 [12,13]. Nop17 binds to the Hsp90-Tah1 complex and has been proposed to control Hsp90 ATPase activity [13]. The structures of the interaction regions of Tah1-Hsp90 and Tah1-Nop17 have been determined, and a model was proposed, according to which the Tah1 TPR domain adopts a highly folded structure, whereas the C-terminal region of Tah1 only folds upon its interaction with Nop17 [14].

Nop17 interacts with proteins involved in various cellular processes [8,15,16], probably helping the assembly of different complexes. The role played by Nop17 in snoRNP assembly depends on its interaction with Hsp90 as part of the R_2_TP complex. Despite the studies on the interactions between the R_2_TP complex and Hsp90, and the determination of the structure of the complex, the molecular function of Nop17 remains elusive.

Rsa1, although not a subunit of the R_2_TP complex, has also been shown to be involved in box C/D snoRNP formation through its interaction with Snu13 and with Nop17 [17]. It has been proposed that Rsa1 binds immature snoRNP particles and is released upon assembly of the mature protein subunits of the complexes for their active conformation [18].

R_2_TP also interacts with the prefoldin complex, which participates in protein folding, degradation and rearrangements [19], broadening the range of protein interactions of the R_2_TP complex, and therefore, of Nop17. In this work, we describe further studies on the interactions between Nop17 and the R_2_TP complex, and between Nop17 and the box C/D snoRNP core subunits. Through the analysis of the interaction of a Nop17 point mutant with Tah1, we were able to narrow down the interface regions of these proteins, and also analyzed the effect of the presence of ATP on the interaction of the Rvb1/2 ATPase with Nop17. In addition, we mapped the region of Nop58 involved in the interaction with Nop17. Based on the data presented here, we propose a model for the role of R_2_TP in snoRNP assembly.

## Results

### Interactions of Nop17 within R_2_TP complex

**Figure 1:**
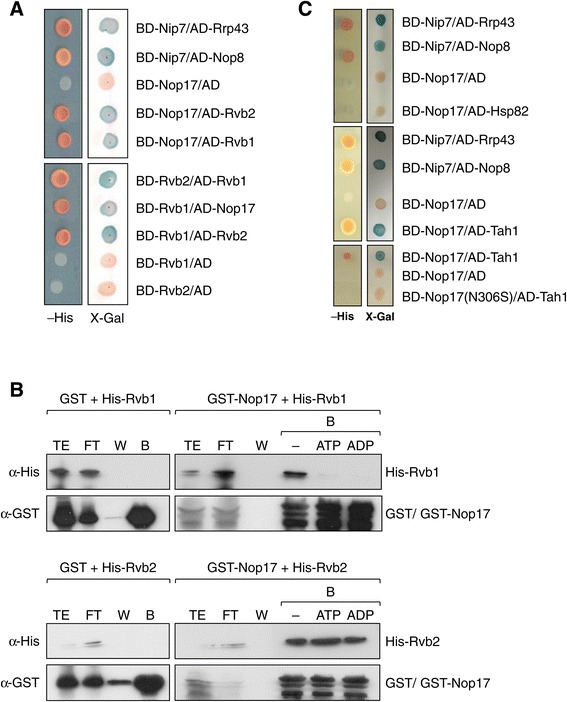
**Interaction of Nop17 with the other subunits of the R**_**2**_ **TP complex. (A)** Analysis of the interactions between Nop17 and Rvb1, and Rvb2 through the two-hybrid assay. BD-Nop17 interacts with both AD-Rvb1 and AD-Rvb2, as seen by the expression of the reporter genes *HIS3* and *lacZ*. BD-Rvb2 + AD-Nop17 is stronger than BD-Rvb1-AD-Nop17. BD-Nip7/AD-Rrp43 and BD-Nip7/AD-Nop8 were used as positive controls for interaction [21,22] **(B)** Pull-down assay to confirm direct interaction between Nop17 and Rvb1/2. GST or GST-Nop17 were bound to glutathione-sepharose beads, followed by the incubation with His-Rvb1, or His-Rvb2, in the absence, or presence of 1 mM ATP or ADP at 4°C for 2 hours. Fractions from total extract (TE), flow through (FT), wash (W), or bound **(B)** were separated by SDS-PAGE and subjected to western blot with anti-His or anti-GST sera. Interaction Nop17-Rvb2 is independent of ATP. **(C)** Two-hybrid assay for the analysis of Nop17-Tah1 and Nop17-Hsp90 interaction. BD-Nop17 did not interact with AD-Hsp90, whereas BD-Nop17 interacted with AD-Tah1. Mutation of Nop17 in the position 306 disrupts interaction with Tah1.

Nop17/Pih1 was identified as part of the R_2_TP complex, together with Rvb1, Rvb2, and Tah1 [20]. In this complex, Nop17 has been shown to interact directly with Tah1 in pull-down assays [9], and with Rvb1 and Rvb2 in the two-hybrid system [17]. In order to analyze in more detail Nop17 interactions with R_2_TP subunits, two-hybrid and pull-down assays were performed. Nop17 showed interaction with Rvb1 and Rvb2 in the two-hybrid system when fused to both domains: the lexA DNA binding domain, and the Gal4 transcription activation domain (Figure 1A). Interaction between Rvb1 and Rvb2 was also positive in both fusions. As expected, Rvb1 and Rvb2 show higher affinity for each other in the two-hybrid assay than for Nop17 (Figure 1A). Since Rvb1 and Rvb2 are ATPases [12], to determine whether ATP binding or hydrolysis may affect the interaction between these proteins and Nop17, pull-down assays were performed with recombinant proteins in the absence or in the presence of either ATP or ADP. The results show that the interaction Nop17-Rvb2 is independent of ATP or its hydrolytic product, whereas Nop17 only interacts efficiently with Rvb1 in the absence of ATP (Figure 1B). These results are interesting because they suggest that Nop17 may interact directly with Rvb2, independently of Rvb1 or ATP. Despite the direct binding of Nop17 to Rvb1, this interaction is hindered by the presence of ATP or ADP.

Experiments with deletion mutants of Nop17 have shown that its C-terminal portion is important for the interaction with Tah1 [23]. To further analyze the region of Nop17 responsible for its interaction with Tah1 and other proteins, we performed random *in vitro* mutagenesis in *NOP17* gene fused to lexA DNA binding domain and tested the interaction of the mutants with Gal4AD-Tah1 in the two-hybrid system. A *nop17* mutant was obtained that no longer interacts with Tah1, this mutant has an asparagine to serine substitution in position 306 (N306S) (Figure 1C). Western blot results show that BD-nop17(N306S) is expressed in L40 cells, although in lower levels than BD-Nop17 (Additional file 1: Figure S1). Interestingly, a very recent report on the structure of Nop17-Tah1 interaction domains show that N306 is part of a beta sheet in Nop17 CS domain, interacting with Tah1 C-terminal region [24]. Further studies will reveal how this mutation might affect Nop17 structure in order to disrupt the interaction with Tah1. Molecular modeling analysis suggests that the asparagine to serine substitution in the position 306 might affect the intramolecular interactions in the Nop17 CS domain (data not shown).

### Rsa1 and Tah1 affect Nop17 stability

**Figure 2:**
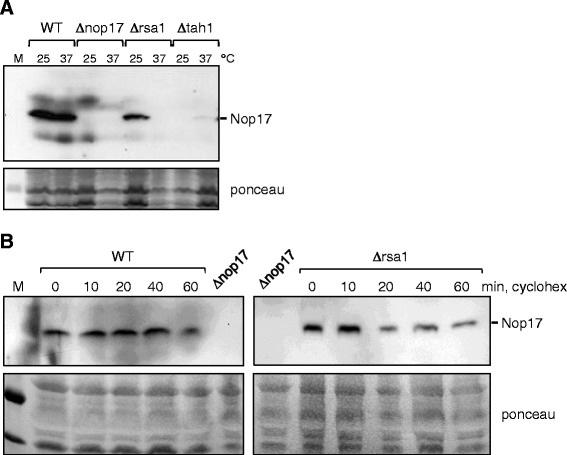
**Absence of Rsa1 or Tah1 destabilizes Nop17. (A)** Total extract from cells growing either at the permissive (25°C), or restrictive (37°C) temperature to OD_600_ 0.5 were used for western blot with serum against Nop17. Steady state levels of Nop17 do not change in wild type cells, whereas in Δ*rsa1* and Δ*tah1* strains, Nop17 levels decrease drastically. **(B)** WT and Δ*rsa1* cells were treated with cyclohexamide after incubation to OD_600_ of 0.8 at 37°C. Samples were collected at the indicated time points and subjected to western blot for the detection of Nop17. Ponceau staining of the membranes was used as control for total protein loaded on gels.

Previous studies have shown that Tah1 interaction is important for Nop17 stability [20]. We therefore tested the expression levels of Nop17 in the Δ*tah1* strain. In addition, due to Rsa1 involvement in snoRNP formation [17,18], we also tested Nop17 in the Δ*rsa1* strain. Since the strains Δ*nop17* and Δ*rsa1* are temperature sensitive, we tested Nop17 levels at the permissive and restrictive growth temperatures. The results show that Nop17 levels decrease in Δ*rsa1* at 25°C, and are even lower at 37°C (Figure 2A). Interestingly, in Δ*tah1* strain, Nop17 levels are very low, regardless of the temperature of growth, suggesting that the interaction with Tah1 is more important for the stability of Nop17, and also suggesting that Nop17 may not be found in the cell in a free form, but only bound to Tah1, either in the R_2_TP complex, or in a ternary complex with Hsp90. Further confirming that the interaction with Tah1 is important for Nop17 stability, the steady-state level of the mutant Nop17(N306S) is lower than that of the wild type protein (Additional file 1: Figure S1). Interestingly, deletion of Rsa1 also leads to the destabilization of Nop17 (Figure 2B). Nop17 shows a half-life of 90 min in WT cells, but it decreases to 55 min in Δ*rsa1* strain at 37°C. These results show that Rsa1 also plays a role in Nop17 stability.

### Nop17 is important for Nop58 stability

**Figure 3:**
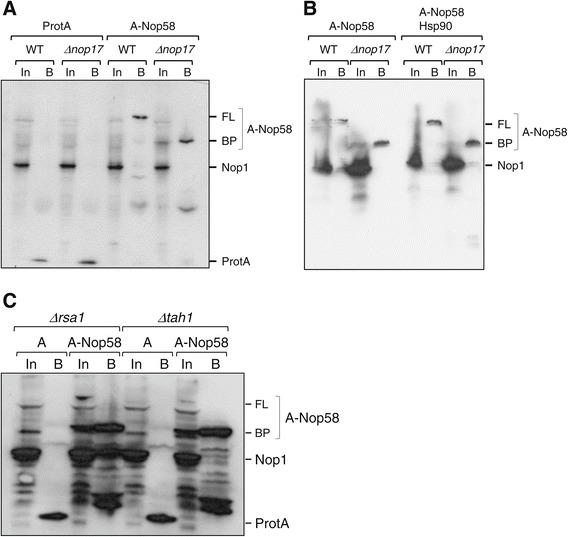
**Nop17, Tah1 and Rsa1 affect Nop58 stability.** Total extract from cells expressing either ProtA or ProtA-Nop58 were used in immunoprecipitation assays. Samples from input (In) and bound **(B)** material were analyzed by western blot for the detection of ProtA-Nop58. **(A)** Expression of ProtA or ProtA-Nop18 in strains WT and Δ*nop17*. Band corresponding to full-length (FL) ProtA-Nop58 can only be detected in samples from WT cells. In samples from Δ*nop17*, break-down products (BP) from ProtA-Nop58 are visualized. Nop1 was used as an internal control for input samples and was not co-immunoprecipitated with ProtA-Nop58 under the conditions used. **(B)** The same experiment as in **A** was performed with samples from cells overexpressing Hsp90. **(C)** Immunoprecipitation of ProtA or ProtA-Nop58 expressed in Δ*rsa1* or Δ*tah1*. Depletion of Rsa1 or Tah1 also destabilizes Nop58.

Nop17 interacts directly with Nop58 and is important for the assembly of the box C/D snoRNP particle, probably by directing Hsp90 chaperone to the particle [8,18]. Considering Δ*nop17* temperature sensitivity, and the involvement of Nop17 in targeting Hsp90-Tah1 to client proteins, those client proteins might be destabilized in the absence of Nop17 at higher temperatures. To test this hypothesis, ProtA-Nop58 levels were assessed in Δ*nop17* strain and compared to wild type cells. Nop58 has been shown to be unstable *in vitro* [3,25], therefore, in order to detect the protein, immunoprecipitation was performed using IgG-sepharose beads. The results show that full-length ProtA-Nop58 can be visualized in the bound fraction from wild type cells, but the protein is destabilized in Δ*nop17* strain, resulting in breakdown products that are detected in the bound fractions (Figure 3A). To test whether higher levels of the chaperone could stabilize Nop58, Hsp90 was overexpressed together with Nop58 in either wild type or Δ*nop17* strains. The results show that in the absence of Nop17, Nop58 is unstable, regardless of the overexpression of the chaperone (Figure 3B), suggesting that indeed Nop17 is required for directing Hsp90 to its client protein Nop58. These data are also in agreement with the model of Nop17 being an Hsp90 co-chaperone, responsible for inhibiting its ATPase activity, which is important for the loading of client proteins onto Hsp90 [13]. Accordingly, loss of Tah1 and Rsa1 has the same destabilizing effect on Nop58 seen in Δ*nop17* strain (Figure 3C). The effects of these latter proteins on Nop58 could be indirect, and depend upon Nop17 interaction with Nop58. These results corroborate the hypothesis of Nop17 being important for directing Hsp90 to Nop58, and thereby, to the box C/D snoRNP complex.

### Nop17 and Rsa1 affect the interaction between Nop58 and U3 snoRNA

**Figure 4:**
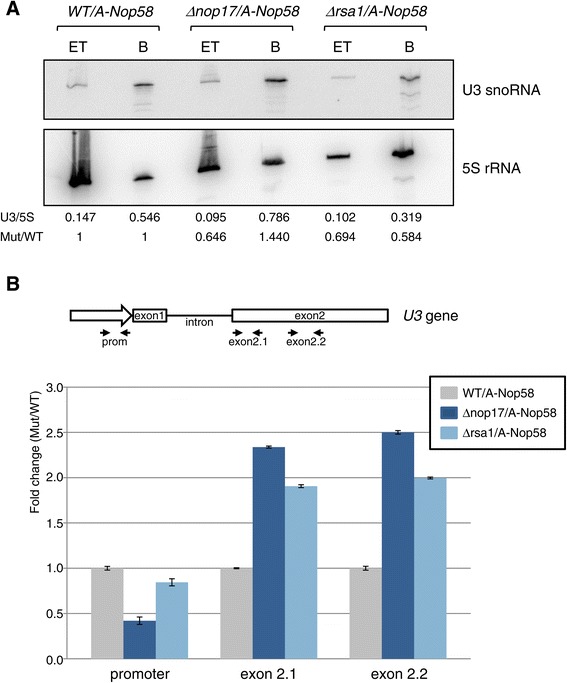
**Co-immunoprecipitation of U3 snoRNA with ProtA-Nop58 in** Δ***nop17*** **cells. (A)** Total cell extracts from strains WT, Δ*nop17* and Δ*rsa1* were mixed with IgG-Sepharose beads for co-immunoprecipitation of snoRNAs with ProtA-Nop58. Bound RNA was detected by northern blotting using probes specific to the snoRNA U3. Membrane was washed and re-hybridized against probe specific to the 5S rRNA (internal control). TE, total extract; B, bound fraction. Bands were quantitated using Typhoon equipment and mean values of three biological replicates are shown. U3 bands were quantitated relative to 5S bands in each lane. U3/5S in mutants were then calculated relative to WT strain. **(B)** Nop58 immunoprecipitates snoRNA U3 chromatin. ChIP assay with A-Nop58 expressed in strains WT, Δ*nop17* and Δ*rsa1* was performed, followed by RT-qPCR reactions with primers for amplification of various regions of the U3 snoRNA gene. Mean values are based on three different experiments with two biological replicates.

Interestingly, despite being less stable upon depletion of the R_2_TP complex, Nop58 shows higher affinity for box C/D snoRNA in the absence of Nop17 [8]. A similar, though weaker, effect is seen in the absence of Rsa1 (Figure 4A), suggesting that the recruitment of Hsp90 and its co-chaperones is important for the correct binding of Nop58 to the snoRNAs. A decrease in the stability of Nop58-snoRNA interaction might be required for the assembly of the other box C/D core subunits onto the complex.

To determine whether Nop58 binds snoRNA at an early or late stage of snoRNP assembly, ChIP assays were performed with different pairs of primers for the analysis of three regions of the U3 snoRNA gene. The results show that Nop58 binds U3 snoRNA co-transcriptionally in the exon 2 region, where the conserved C/D are located (Figure 4B). Corroborating the previous results, Nop58 bound snoRNA with higher affinity upon depletion of either Nop17 or Rsa1 (Figure 4B).

**Figure 5:**
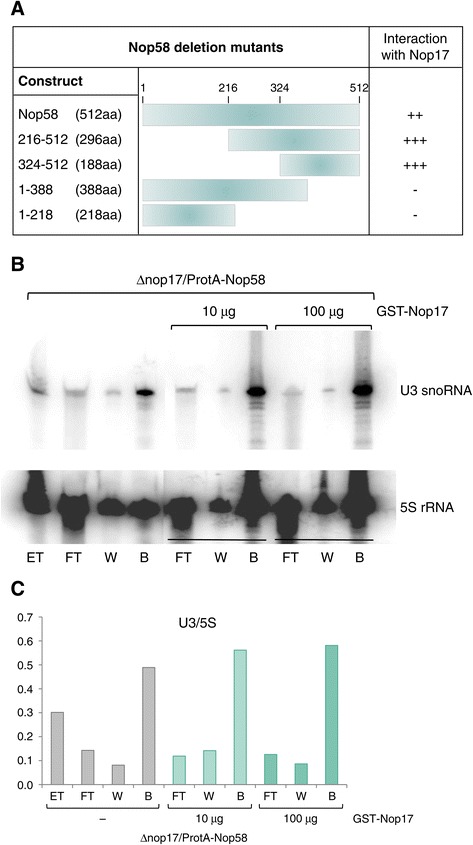
**Nop58 interacts with Nop17 through its C-terminal portion. (A)** Schematics summarizing the results of two-hybrid assay with deletion mutants of Nop58 and Nop17. Nop58(324–512) mutant interacts with Nop17. **(B)** Co-immunoprecipitation of RNA with ProtA-Nop58 in the absence or presence of different amounts of Nop17. Incubation of total extract with IgG-sepharose beads was performed for 2 h at 4°C in the presence or absence of purified GST-Nop17. Co-immunoprecipitated U3 snoRNA was detected by northern blot. 5S rRNA was used as an internal control. **(C)** Quantitation of the U3 bands corrected by 5S bands after northern hybridization.

The structure of the archaeal complex Nop58/Nop1 orthologues has been determined [26]. From the structure of the archaeal complex and the analysis of protein interactions with RNA, it has been suggested that the C-terminal region of Nop5 is involved in the interaction with RNA and the protein L7Ae, the central coiled-coil region is important for the Nop5 dimerization, while the N-terminal portion interacts with Fibrillarin [27]. Taking into account the protein sequence conservation and the complexes formed in archaea and eukaryotes, the information from the archaeal complex can be used to infer that the C-terminal portion of Nop58 is responsible for its interaction with snoRNAs. As shown here and previously, Nop58 co-immunoprecipitates box C/D snoRNAs more efficiently in the absence of Nop17 or Rsa1 (Figure 4) [8], suggesting that Nop17, which interacts directly with Nop58, might compete with RNA for the interaction with the C-terminal portion of Nop58. We therefore used the two-hybrid assay to map the region of Nop58 involved in the interaction with Nop17. The results show that Nop17 interacts with the C-terminal region of Nop58, but not with the N-terminal, or the central domains of Nop58 (Figure 5A; Additional file 2: Figure S2). Although we cannot exclude the possibility that N-terminal portions of Nop58 may not interact with Nop17 due to lower protein levels, we consider that unlikely because the region of Nop58 responsible for its instability is a highly charged KKD/E domain located at its C-terminal portion [25]. Our results therefore suggest that either Nop17 can compete with RNA for binding to Nop58, or that upon interacting with Nop17, Nop58 has its affinity for RNA decreased. To test the hypothesis of Nop17 competing with RNA for binding Nop58, we performed RNA co-immunoprecipitation assays with ProtA-Nop58 expressed in Δ*nop17* strain, and added increasing amounts of recombinant GST-Nop17 during the assay. The results show that the snoRNA U3 co-purified with ProtA-Nop58 is not released in the presence of the purified Nop17 (Figure 5B,C). These results suggest that Nop17 does not compete with RNA for binding Nop58 *in vitro*. It is also possible that only the complete R_2_TP complex may affect Nop58-RNA interaction.

### Nop17 interacts with other box C/D snoRNP subunits in addition to Nop58

**Figure 6:**
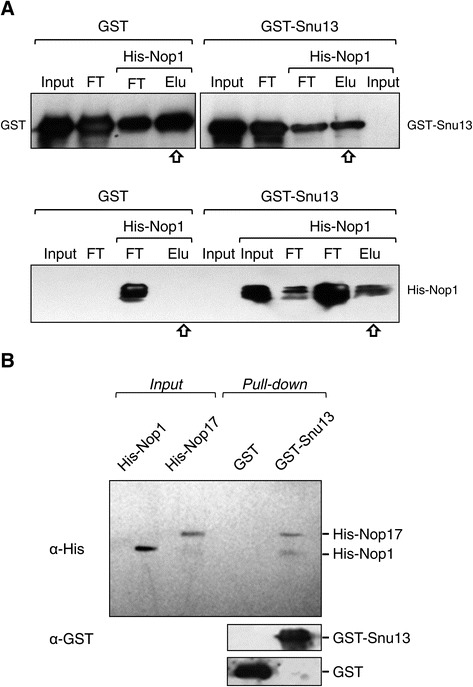
**Interaction between Nop17 and other C/D box snoRNP subunits. (A)** Protein pull-down to visualize the interaction between Nop1 and Snu13. Total extracts from *E. coli* cells expressing GST or GST-Snu13 (input) were first incubated with GST-sepharose beads. Extracts from cells expressing His-Nop1 (input) were then added to the beads. Flow through was collected (FT) after the addition of each extract, and beads were washed. Proteins were eluted with reduced glutathione (Elu). Samples from each fraction were subjected to SDS-PAGE and western blot with anti-GST and anti-His sera. Elution fractions are indicated by arrows. **(B)** Nop17 interacts with the complex Nop1/Snu13 in pull-down assays. His-Nop1 was added to either GST or GST-Snu13 immobilized in glutathione-sepharose beads. After that, His-Nop17 was added to the beads, and was pulled-down only by the GST-Snu13/His-Nop1 complex. Bands were visualized by western blot with anti-GST and anti-His sera.

We have previously shown that Snu13 interacts with all the other three core subunits of box C/D snoRNPs, but it does not interact with Nop17 in the two-hybrid system [8]. To determine the snoRNP assembly step in which Nop17 is involved, we performed protein pull-down experiments with recombinant Snu13, Nop1 and Nop17. In these experiments, His-Nop1 is efficiently pulled down with GST-Snu13, but not with GST (Figure 6). Interestingly, His-Nop17 can be co-precipitated with His-Nop1 when the latter is bound to GST-Snu13 (Figure 6B). These results suggest that Nop17 can bind the heterodimer Snu13-Nop1, but not the isolated proteins. This can be an indication that Nop17 interacts with these two proteins after they bind the snoRNA, already as part of the snoRNP assembly complex. Nop17 might be brought to the complex by its interaction with Nop58, decreasing the affinity of Nop58 for the snoRNA and allowing for the assembly of Nop1 and Snu13 onto the complex. The hydrolysis of ATP by Rvb1/2 ATPases may cause a structural rearrangement necessary for the release of the R_2_TP complex and formation of the mature box C/D snoRNP.

### Nop17 and Rsa1 affect U3 snoRNA localization

**Figure 7:**
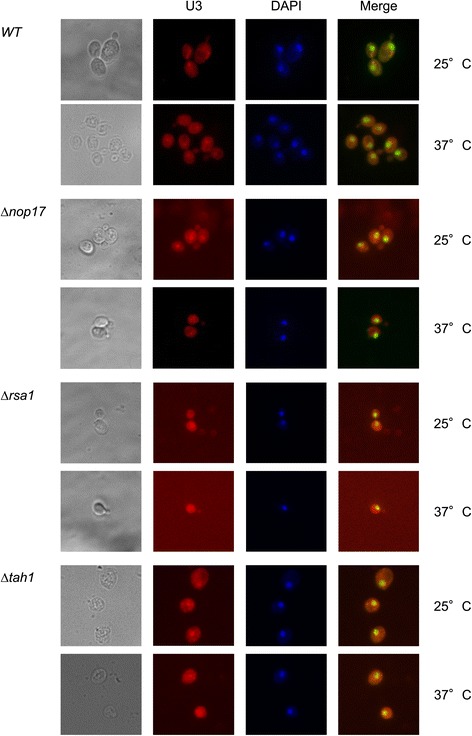
**Depletion of R**_**2**_ **TP subunits affects the localization of the U3 snoRNA.** FISH experiments were performed using cells that had been cultivated at 25°C or 37°C before hybridization with a fluorescent-labeled DNA oligo complementary to U3 snoRNA. DNA was labeled with DAPI.

The role of R_2_TP, and particularly of Nop17, in the assembly of box C/D snoRNP suggests that there might be an order of binding of the proteins to the snoRNA and molecular rearrangements for the formation of the mature complex. Therefore, the depletion of the R_2_TP subunits might not only cause a mislocalization of the box C/D proteins, as has been demonstrated for Nop17 [8], but also of the snoRNAs. To test this hypothesis, we performed FISH experiments to determine the U3 snoRNA localization in the deletion strains Δ*nop17*, Δ*rsa1*, and Δ*tah1*, compared to the wild type strain growing at the permissive or non permissive temperature. The results show that in the absence of Nop17 or Rsa1, U3 snoRNA is still concentrated in the nucleolus at 25°C, but when the cells are shifted to 37°C, the snoRNA signal becomes more disperse throughout the cells (Figure 7; Additional file 3: Figure S3). Surprisingly, however, depletion of Tah1 did not seem to affect the localization of U3 snoRNA. These results indicate that in the absence of Nop17 or Rsa1, the assembly of box C/D snoRNP is defective, mainly at the non-permissive temperature, leading to the mislocalization of the snoRNA. Interestingly, as pointed out above, absence of Nop17 has the same effect on the box C/D core proteins [8], confirming the importance of this protein for the assembly of snoRNP. Because the localization of U3 snoRNA is not strongly affected by the depletion of Tah1, these results also suggest that its function might be redundant with that of another Hsp90 co-chaperone.

## Discussion

Nop17/Pih1 is a nucleolar protein [8] that has been shown to be part of the R_2_TP complex, interacting with Rvb1, Rvb2, and Tah1 [9]. Rvb1 and Rvb2 are ATP dependent helicases thought not to be present in isolated form in the cell, but instead they may form a heterohexameric complex containing three molecules of each protein [12]. This complex shows higher helicase activity *in vitro* than the isolated proteins, and undergoes nucleotide-dependent conformational changes [11,12]. Based on the results shown here, it can be hypothesized that Nop17 binds more tightly to one of the conformations of Rvb1/Rvb2 complex, thereby modulating the activity of the complex.

Nop17 has been shown to interact with Tah1 through its C-terminal region [13,14,28], it was therefore important to determine whether point mutations in the C-terminus of Nop17 affect its interaction with Tah1. As shown here, substitution in the position 306 of Nop17 abolishes its interaction with Tah1. Interestingly, these results are corroborated by the recent determination of the structure of the Nop17-Tah1 interaction domains [24]. Amino acid 306 is part of a beta sheet in the interaction pocket of Nop17 with Tah1. The amide to OH change in the N306S mutant might disrupt the interaction with the C-terminal segment of Tah1 [24]. Whereas the C-terminal region of Tah1 interacts with Nop17, its N-terminal TPR domain interacts with Hsp90 C-terminal peptide MEEVD [28]. Tah1-Hsp90 interaction might be stabilized by the interaction between Nop17 and Hsp90 [20].

As shown here, Tah1 is important for the stability of Nop17, this stabilization may be due to the interaction with the C-terminal region of Nop17. Similar results have been shown for the human orthologues of these proteins [29]. The destabilizing effect on Nop17 was not seen, however, when a Nop17-FLAG fusion was used, probably due to the protein fusion [20].

Tah1 is specific for Hsp90 and affects its ATPase activity as well as substrate binding [30]. It is interesting, therefore, that in the absence of either Nop17 or Tah1 the box C/D core subunit Nop58 is destabilized. More importantly, as shown here, the depletion of Rsa1, a protein proposed to function as a scaffold for snoRNP assembly [31], also leads to the destabilization of Nop58, confirming the importance of the R_2_TP interaction with Hsp90 and Rsa1 for the proper assembly of the functional snoRNP complex.

**Figure 8:**
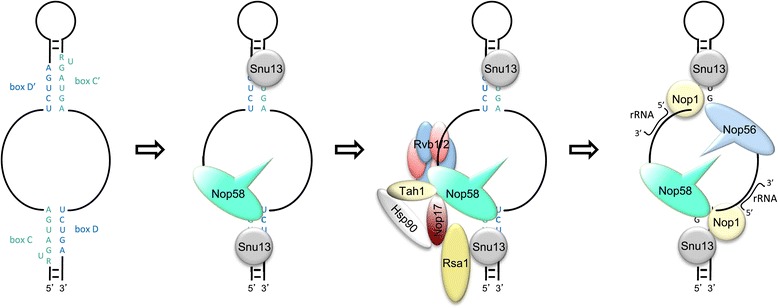
**Model of the role of the R**_**2**_ **TP complex in the assembly of box C/D snoRNP.** Snu13 binds early during the assembly, and Nop58 binds the snoRNA cotranscriptionally. The association of the R2TP complex and Hsp90 chaperone are important for the conformational change of Nop58, necessary for the binding of the Nop1 and Snu13 to form the mature snoRNP particle that will participate in rRNA maturation. In the absence of R_2_TP subunits, Nop58 is less stable, but shows higher affinity for RNA. In addition, in the absence of R2TP, snoRNAs and core snoRNP subunits mislocalize in the cell.

According to the model of Rsa1 being a scaffold for the snoRNP assembly [31], Nop58 would bind the snoRNAs only after it is directed to the box C/D particle by the R_2_TP complex. As shown here, however, Nop58 binds U3 snoRNA co-transcriptionally, and binds snoRNA more stably in the absence of Nop17 [8]. We, therefore, favor a model according to which Nop58 binds snoRNA with very high affinity, but in order for the snoRNP complex to be matured, this interaction must be destabilized so that Nop58 can interact with Nop56 and allow for Nop1 to bind box D and D’ (Figure 8). The structure of the PIH domain of hNop17/PIH1D1 interacting with a target peptide DSDD has been determined, and it has been shown that this interaction is dependent upon the phosphorylation state of the peptide DSDD [32]. Interestingly, Nop58 has this conserved peptide in the C-terminal portion involved in the interaction with Nop17 (positions 443–446). It remains to be determined whether the serine 444 is phosphorylated, and whether its phosphorylated state changes upon snoRNP assembly.

The model for box C/D snoRNP assembly proposed here is also supported by the observation that core box C/D snoRNP subunits have been shown to be important for snoRNA localization [33]. Further corroborating this hypothesis, the depletion of Nop17 or Rsa1 causes a mislocalization of the snoRNA U3.

During the final preparation of this article a study was published on the R_2_TP complex [34]. That study reports the increased interaction of R_2_TP with Nop58 in the absence of RNA, and the importance of R_2_TP on Nop58 stability. In that report the Nop58 regions involved in the interaction with Nop17 were also mapped. In addition, they showed that the dissociation of Rvb1/2 from Nop17/Tah1 was induced by nucleotide binding rather than ATP hydrolysis. Those results are consistent with the data shown here. We show complementary data of the stronger interaction between Nop58 and RNA in the absence of R_2_TP. Additionally, we show that Nop17 does not compete with RNA for binding to Nop58, confirming that RNA is not necessary for the Nop17-Nop58 interaction. The data shown here also complement those because we narrow further down the region of Nop58 responsible for the interaction with Nop17. As shown here, that region is enclosed between amino acids 389 and 512. We can therefore conclude that the 389–447 portion of Nop58 is responsible for that interaction. Interestingly, this region comprises the peptide DSDD, recently shown to interact directly with the PIH domain of Nop17 [32].

We show that the levels of full-length Nop58 decreases upon depletion not only of Nop17, but also of Rsa1 and Tah1. Interestingly, and according to the model of Nop17 directing Hsp90 to the target proteins, as shown here, the over-expression of Hsp90 in Δ*nop17* strain does not stabilize Nop58. Here we show that the interaction between Nop17 and Rvb2 is not affected by nucleotide binding or hydrolysis, contrary to the interaction with Rvb1 which is affected by the presence of ATP. Our results, therefore, corroborate and complement those recently published.

## Conclusions

Nop17 interacts with the C-terminal portion of Nop58, affecting its affinity for snoRNA, Nop58 stability, and the localization of the box C/D snoRNP components, both protein and RNA moieties. Tah1 and Rsa1 affect Nop17 stability, and might therefore affect Nop58 indirectly. These results indicate a key role played by Nop17 in snoRNP assembly, and suggest a stepwise process that requires molecular rearrangements of the proteins for the binding of all subunits and formation of the mature snoRNP.

## Methods

### Plasmid constructions

The plasmids used in this study are listed on Table 1 and the cloning strategies described below. The genes RVB1, RVB2 and HSC82 were amplified from *S. cerevisiae* genomic DNA. For recombinants proteins with expression in bacteria (fused to glutathione-S-transferase (GST) and histidine tag (His_6_)), the PCR products (RVB1 and RVB2) were first cloned into pGEM-T vector before digestion with EcoRI and SalI for cloning into the vectors pET28a and pGEX-4 T-1. The construct pGEX-NOP17 was obtained after excision of NOP17 from pET28a-NOP17 [8] with BamHI and XhoI. The genes NOP1 and SNU13 were excised from pGAD clones [8] for subcloning into the PET28a and pGEX-4 T-1 vectors. For recombinant proteins expressed in yeast (in fusion with GAL4 and LEXA), pBTM116 and pGAD-C2, RSA1, TAH1, RVB1 and RVB2 were subcloned from pGEM-T. For Hsc82 overexpression, HSC82 PCR product was digested with BamHI and XhoI and cloned into the YCP-111-GAL-HA construct [35].

**Table 1:** **Plasmids used in this study**

**Plasmid**	**Characteristics**	**Reference**
pGEX4T1	GST Tag C-terminal, Amp^R^	Amersham
pGEX4T1-SNU13	GST::SNU13, Amp^R^	This study
pGEX4T1-NOP17	GST::NOP17, Amp^R^	This study
pET28a	His Tag N-terminal, Kan^R^	Novagen
pET28a-SNU13	HIS::SNU13, Kan^R^	This study
pET28a-NOP1	HIS::NOP1, Kan^R^	This study
pET28a-NOP17	HIS::NOP17, Kan^R^	Gonzales et al. [8]
pBTM116	lexA, DNA binding domain	Chien et al. [36]
pBTM-NOP17	lexA::NOP17, TRP1	Gonzales et al. [8]
pBTM-NOP17(N307S)	lexA::NOP17(N307S), TRP1	This study
pGAD	GAL4AD, transcription activation domain	James et al. [37]
pGAD-TAH1	GAL4AD::TAH1, LEU2	This study
pGAD-HSC82	GAL4AD::HSC82, LEU2	This study
pGAD-RVB1	GAL4AD::RVB1, LEU2	This study
pGAD-RVB2	GAL4AD::RVB2, LEU2	This study
YCP111GAL-HA-HSC82	GAL4AD::HA::Nop58, TRP1	This study
YCP33Gal-A-NOP58	GAL::ProtA-NOP58, URA3, CEN4	Gonzales et al. [8]
pBTM-RVB1	lexA::RVB1, TRP1	This study
pBTM-RVB2	lexA::RVB2, TRP1	This study
pET28a-RVB1	HIS::RVB1, Kan^R^	This study
pET28a-RVB2	HIS::RVB2, Kan^R^	This study

### Analysis of protein stability

Cells were grown at 25°C or 37°C to OD_600_ of 0.8, before addition of cyclohexamide to the final concentration of 150 μg/ml. Samples were collected at time zero, and 10, 20 40 and 60 min after addition of cyclohexamide. Total cell extract was then prepared and protein samples were separated by SDS-PAGE and subjected to western blot.

### Co-immunoprecipitation of proteins and western blot analysis

Extracts from strains *WT, ∆nop17*, *∆rsa1* and *∆tah1* expressing ProtA-Nop58 that had been grown in galactose medium were prepared in buffer (20 mM Tris-Cl pH 8.0; 0.5 mM magnesium acetate; 0.2% Triton X-100; 150 mM potassium acetate; 1 mM DTT and 1 mM PMSF). Immunoprecipitation was performed by incubating total extract with *IgG-Sepharose* (Amersham Biosciences) for 2 hours at 4°C. Fractions corresponding to total extract (TE), flow-trough (FT), wash (W) and immunoprecipitation (IP) were collected and stored at −20°C. For protein analysis by western blot, samples were separated by SDS-PAGE and transferred to PVDF membranes (GE Healthcare). For ProtA-Nop58 and HA-Hsc82 expression in *WT* and *∆nop17*, the same experiment strategy was used.

### Co-immunoprecipitation of RNAs and northern blot

RNA co-immunoprecipitation with ProtA-Nop58 expressed at 37°C in strains *WT*, *∆nop17* and *∆rsa1* was performed as described previously [8]. For the Nop58 and RNA binding assay, co-immunoprecipitation was performed as described [8], and purified GST-Nop17 was added during the incubation of total extract with IgG-sepharose beads during the immunoprecipitation. Northern hybridizations were analyzed in a Typhoon equipment (GE Healthcare Life Sciences) and bands quantitated using ImageQuant program from Typhoon.

### Chromatin immunoprecipitation and qPCR analyses

*WT*, *Δnop17* and Δ*rsa1* cells expressing ProtA-Nop58, were fixed with 1% formaldehyde and used for chromatin immunoprecipitation, as previously described [38]. For the qPCR reactions, the *Maxima SYBER Green/ROX qPCR Master Mix* (Molecular Probes Inc., Eugene) was used in the Real Time Applied Biosystem 7500 equipment. The primers used in the reactions are listed on Table 2.

**Table 2:** **Oligonucleotides used in this study**

**Oligonucleotide**	**Sequence**	**Reference**
5S^rev^	GCGAGGCAAATCCTGAAAATTT	Granato et al. [15]
U3 prom^for^	CGAAGGCAAATCCTGAAAATTT	This study
U3 prom^rev^	TTGACAGCAGAATACAAAGCCTTT	This study
U3 exon1^for^	TCAACCATTGCAGCAGCTTT	Coltri et al. [39]
U3 exon1^rev^	TCTGCTCCGAAATGAAAACTCTAGTA	Coltri et al. [39]
U3 exon2^for^	TCTATAGGAATCGTCACTCTTTGACTC	Coltri et al. [39]
U3 exon2^rev^	GACCAAGCTAATTTAGATTCAATTTCGG	Coltri et al. [39]
U3RevFISH	ATGGGGCTCATCAACCAAGTTGG	This study

### snoRNA localization by FISH

The detailed protocol used for the *in situ* hybridization in strains *WT, ∆nop17*, *∆rsa1* and *∆tah1* is described in http://www.einstein.yu.edu/labs/robert-singer/protocols/. The fluorescence tagging system used was the Cy™3ULS Labeling Kit (Amersham Biosciences) and images were obtained with a Nikon TE300 microscope coupled to a Roper CoolSnap HQ camera. Quantitation of the fluorescence signals was performed using ImageJ 1.42q.

### Recombinant proteins expression in bacteria and pull-down of proteins *in vitro*

Recombinant proteins GST-Snu13, GST-Nop17, His-Nop1, His-Nop17, His-Rvb1 and His-Rvb2 were expressed in *E. coli* strain BL21. For the Snu13-Nop1-Nop17 pull-down, the total extracts containing GST or GST-Snu13 were incubated for 1 hour at 4°C with glutathione-sepharose resin (GE Healthcare) in PBS buffer. After immobilization of the first protein, the resin was washed with buffer and incubated for 2 hours with total extract expressing His-Nop1 or His-Nop17, with three washes between the extracts. After the last wash, the proteins were eluted from the resin with PBS buffer containing 10 mM reduced glutathione. Samples of all fractions were collected (TE, FT, W and Elu), and the proteins were analyzed by western blot with anti-GST, anti-His and anti-Nop17 sera. For the pull-down between Nop17 and Rvb1 and Rvb2, the same strategy was used, with the addition ATP or ADP during the second incubation step.

### Construction of Nop17 mutant

Nop17 mutant was obtained by random *in vitro* mutagenesis [35], using pBTM-NOP17 as a template. Interaction with Tah1 was performed in the two-hybrid system using the L40/pGAD-TAH1 strain. Two-hybrid assay was performed as described previously [35].

## Additional files

Additional file 1: Figure S1. Analysis of expression of mutant Nop17(N306S) in the cells used for two-hybrid assays. Total extracts were prepared from L40 cells, either not transformed with any plasmid (−), or transformed with pBTM-NOP17, pBTM-NOP17(N306S), pGAD-TAH1, and pGAD-RVB1, as indicated, and subjected to western blot with serum against Nop17. Endogenous Nop17, BD-Nop17 and BD-Nop17(N306S) bands are indicated on the right. Endogenous Nop17 levels, used as loading control, do not vary between the samples, but BD-Nop17(N306S) levels are much lower than those of BD-Nop17. Additional file 2: Figure S2. Two-hybrid assay to map the Nop58 interaction domain with Nop17. *HIS3* expression is shown on the left, while *lacZ* expression is shown on the right. Upper panel, full-length Nop58 interacts with Nop17 independently of the protein fusion (DNA binding domain – BD, or transcription activation domain – AD), and interacts also with Snu13. Middle panel, Nop58(216–512) interacts only with Nop17. Lower panel, Nop58(324–512) interacts only with Nop17, but with higher affinity than the full-length protein. Additional file 3: Figure S3. Quantitation of snoRNA U3 signal from FISH experiments shown in Figure 7. U3 signals in linear distribution throughout the cells were quantitated by using ImageJ. Position of the nucleolus in each cell is indicated. WT cells show concentration of U3 signal in the nucleolus, independently of the temperature of growth. Δ*nop17* shows concentration of U3 in the nucleolus at the permissive temperature, but not at the restrictive temperature. Δ*rsa1* and Δ*tah1* cells show very low signal of U3, but despite that, it is possible to see the mislocalization of U3 at 37°C in Δ*rsa1*. In Δ*tah1* cells, on the other hand, U3 localization does not change much at 37°C.

## Abbreviations

snoRNP: Small nucleolar ribonucleoprotein
R_2_TP: Rvb1, Rvb2, Tah1, Pih1
rRNA: ribosomal RNA
