# Supplementary figures and images for: Nop17 is a key R2TP factor for the assembly and maturation of box C/D snoRNP complex
Source: BMC Mol Biol. 2015 Mar 18;16:7. doi: 10.1186/s12867-015-0037-5 (PMC4377001; doi:10.1186/s12867-015-0037-5)

## Slide 1
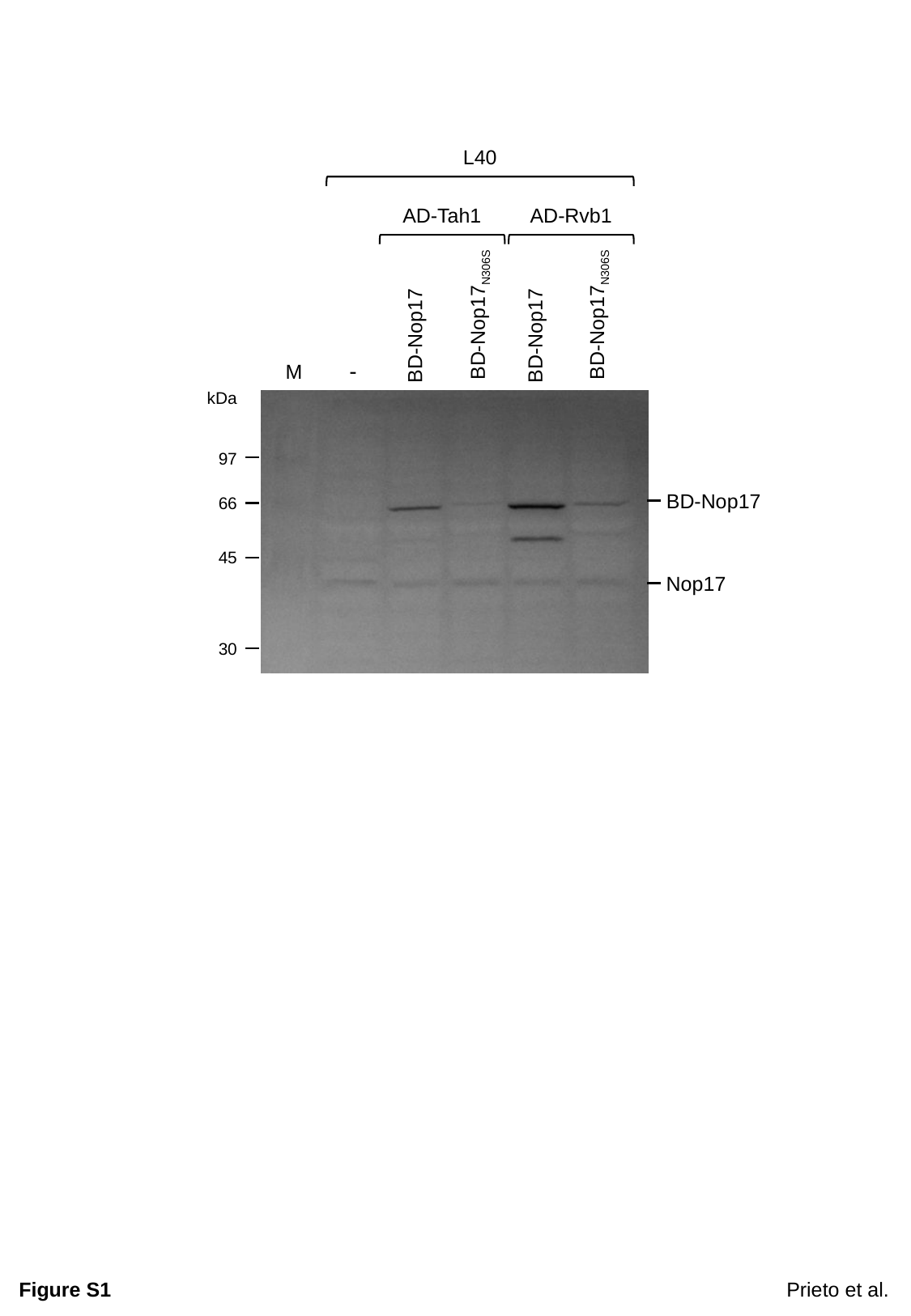

L40
AD-Tah1
AD-Rvb1
BD-Nop17N306S
BD-Nop17N306S
BD-Nop17
BD-Nop17
M
−
kDa
97
BD-Nop17
66
45
Nop17
30
Figure S1
Prieto et al.

Supplement: Additional file 1: Figure S1. — Analysis of expression of mutant Nop17(N306S) in the cells used for two-hybrid assays. Total extracts were prepared from L40 cells, either not transformed with any plasmid (−), or transformed with pBTM-NOP17, pBTM-NOP17(N306S), pGAD-TAH1, and pGAD-RVB1, as indicated, and subjected to western blot with serum against Nop17. Endogenous Nop17, BD-Nop17 and BD-Nop17(N306S) bands are indicated on the right. Endogenous Nop17 levels, used as loading control, do not vary between the samples, but BD-Nop17(N306S) levels are much lower than those of BD-Nop17. [file 12867_2015_37_MOESM1_ESM.pptx]
