# Supplementary material for: Nop17 is a key R2TP factor for the assembly and maturation of box C/D snoRNP complex
Source: BMC Mol Biol. 2015 Mar 18;16:7. doi: 10.1186/s12867-015-0037-5 (PMC4377001; doi:10.1186/s12867-015-0037-5)
Supplement: Additional file 2: Figure S2. — Two-hybrid assay to map the Nop58 interaction domain with Nop17. HIS3 expression is shown on the left, while lacZ expression is shown on the right. Upper panel, full-length Nop58 interacts with Nop17 independently of the protein fusion (DNA binding domain – BD, or transcription activation domain – AD), and interacts also with Snu13. Middle panel, Nop58(216–512) interacts only with Nop17. Lower panel, Nop58(324–512) interacts only with Nop17, but with higher affinity than the full-length protein. [file 12867_2015_37_MOESM2_ESM.pptx]

## Slide 1
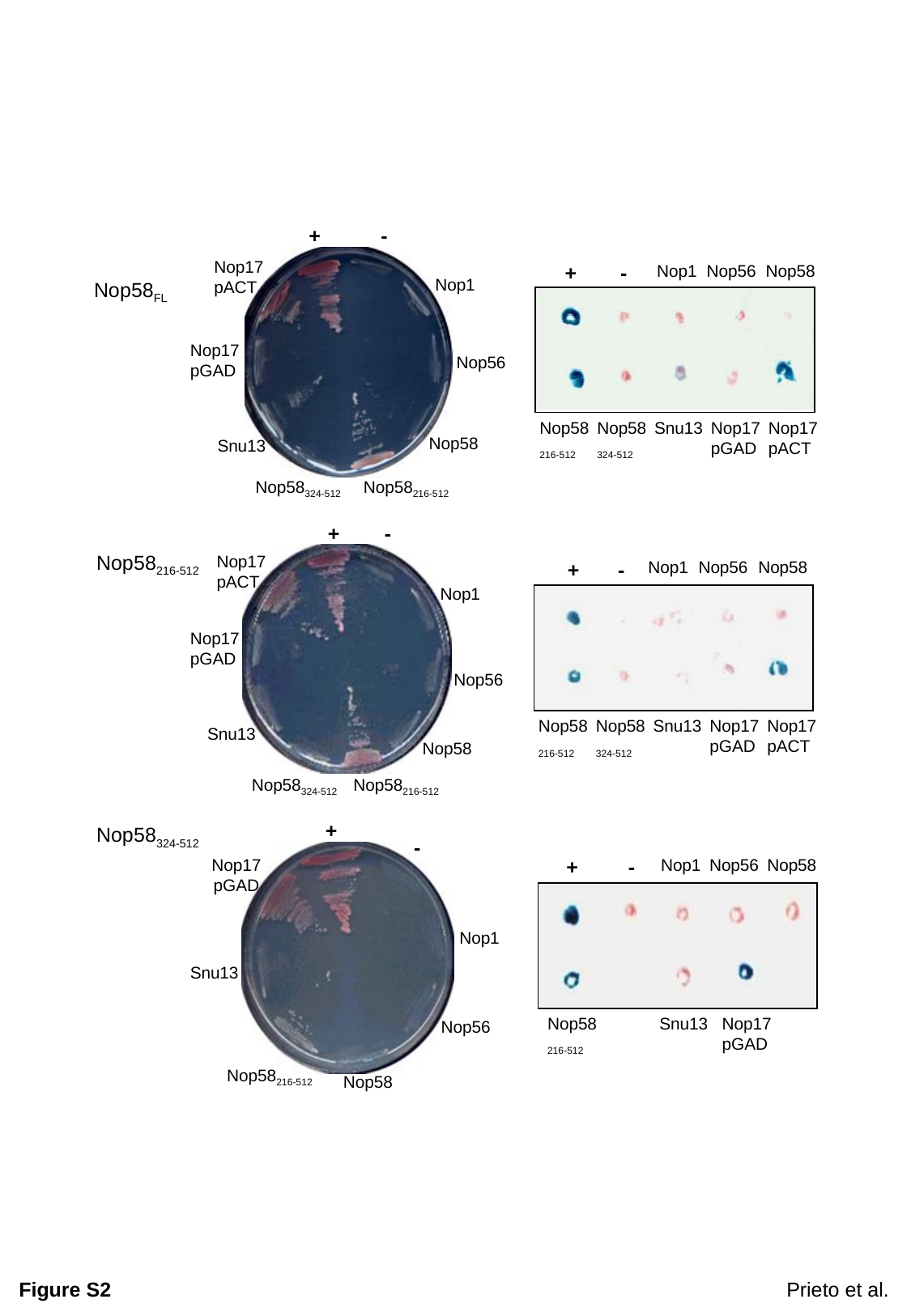

+
-
Nop17
pACT
+
-
Nop1
Nop56
Nop58
Nop1
Nop58FL
Nop17
pGAD
Nop56
Nop58
216-512
Nop58
324-512
Snu13
Nop17
pGAD
Nop17
pACT
Nop58
Snu13
Nop58324-512
Nop58216-512
+
-
Nop58216-512
Nop17
pACT
+
-
Nop1
Nop56
Nop58
Nop1
Nop17
pGAD
Nop56
Nop58
216-512
Nop58
324-512
Snu13
Nop17
pGAD
Nop17
pACT
Snu13
Nop58
Nop58324-512
Nop58216-512
+
Nop58324-512
-
Nop17
pGAD
+
-
Nop1
Nop56
Nop58
Nop1
Snu13
Nop58
216-512
Snu13
Nop17
pGAD
Nop56
Nop58216-512
Nop58
Figure S2
Prieto et al.
