# Supplementary material for: Nop17 is a key R2TP factor for the assembly and maturation of box C/D snoRNP complex
Source: BMC Mol Biol. 2015 Mar 18;16:7. doi: 10.1186/s12867-015-0037-5 (PMC4377001; doi:10.1186/s12867-015-0037-5)
Supplement: Additional file 3: Figure S3. — Quantitation of snoRNA U3 signal from FISH experiments shown in Figure 7. U3 signals in linear distribution throughout the cells were quantitated by using ImageJ. Position of the nucleolus in each cell is indicated. WT cells show concentration of U3 signal in the nucleolus, independently of the temperature of growth. Δnop17 shows concentration of U3 in the nucleolus at the permissive temperature, but not at the restrictive temperature. Δrsa1 and Δtah1 cells show very low signal of U3, but despite that, it is possible to see the mislocalization of U3 at 37°C in Δrsa1. In Δtah1 cells, on the other hand, U3 localization does not change much at 37°C. [file 12867_2015_37_MOESM3_ESM.pptx]

## Slide 1
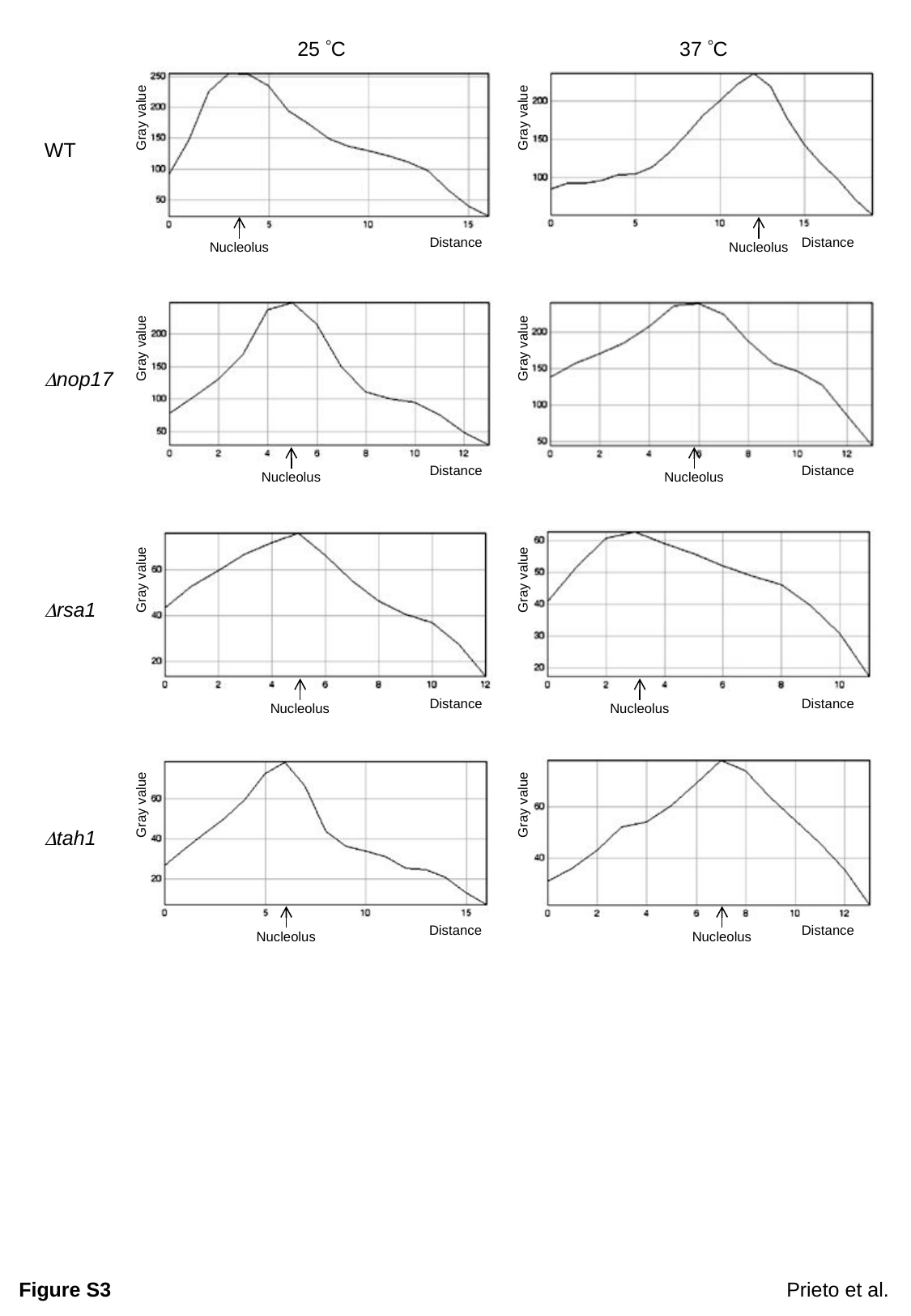

25 °C
37 °C
Gray value
Gray value
WT
Nucleolus
Nucleolus
Distance
Distance
Gray value
Gray value
Δnop17
Nucleolus
Nucleolus
Distance
Distance
Gray value
Gray value
Δrsa1
Nucleolus
Nucleolus
Distance
Distance
Gray value
Gray value
Δtah1
Nucleolus
Nucleolus
Distance
Distance
Figure S3
Prieto et al.
